# Iron Metabolism in Cancer and Senescence: A Cellular Perspective

**DOI:** 10.3390/biology12070989

**Published:** 2023-07-11

**Authors:** Elvira Crescenzi, Antonio Leonardi, Francesco Pacifico

**Affiliations:** 1 Istituto per l’Endocrinologia e l’Oncologia Sperimentale, CNR, Via S. Pansini, 5, 80131 Naples, Italy; e.crescenzi@ieos.cnr.it; 2 Dipartimento di Medicina Molecolare e Biotecnologie Mediche, “Federico II” University of Naples, Via S. Pansini, 5, 80131 Naples, Italy; leonardi@unina.it

**Keywords:** iron, cancer, senescence

## Abstract

**Simple Summary:**

Iron is an essential element in human cells. Cells use iron for many processes, such as proliferation, survival, DNA synthesis and energy production. However, iron overload is dangerous and can cause damage to cells. Hence, iron metabolism and balance are tightly regulated in order to avoid iron accumulation or iron depletion. Studies published in recent years have demonstrated that iron metabolism is dysregulated in cancer cells and that such alterations help the tumor to grow, invade and survive anticancer therapies. For these reasons, iron represents a potential useful target for cancer therapy. The dysregulation of iron metabolism has also been observed in senescent cells, but in this case, our knowledge is still expanding. In this review, we first provide an overview of iron metabolism and iron regulatory proteins. Then, we summarize what we currently know about iron balance in cancer cells and senescent cells.

**Abstract:**

Iron participates in a number of biological processes and plays a crucial role in cellular homeostasis. Alterations in iron metabolism are considered hallmarks of cancer and drivers of aggressive behaviors, such as uncontrolled proliferation, resistance to apoptosis, enhanced metastatic ability, increased cell plasticity and stemness. Furthermore, a dysregulated iron metabolism has been associated with the development of an adverse tumor microenvironment. Alterations in iron metabolism have been described in cellular senescence and in aging. For instance, iron has been shown to accumulate in aged tissues and in age-related diseases. Furthermore, in vitro studies demonstrate increases in iron content in both replicative and stress-induced senescent cells. However, the role, the mechanisms of regulation and dysregulation and the effects of iron metabolism on senescence remain significantly less characterized. In this review, we first provide an overview of iron metabolism and iron regulatory proteins. Then, we summarize alterations in iron homeostasis in cancer and senescence from a cellular point of view.

## 1. Introduction

### 1.1. Iron Metabolism in Normal Cells

Iron is a transition element that is abundantly present on the surface of our planet [1]. What makes iron unique, particularly in living organisms, is its ability to donate and accept electrons in redox reactions [2]. In this way, iron actively participates in a number of biological processes such as DNA synthesis and repair, mitochondrial cellular respiration, cell proliferation, cell survival, cell signaling and host defense [3]. The majority (80%) of the iron in the human body is incorporated into the heme of erythrocyte hemoglobin to ensure oxygen transport along the bloodstream, while the remaining 20% can mainly be found in macrophages and hepatocytes bound to ferritin or in myoglobin and iron-containing enzymes in various cell types [4]. 

**Figure 1:**
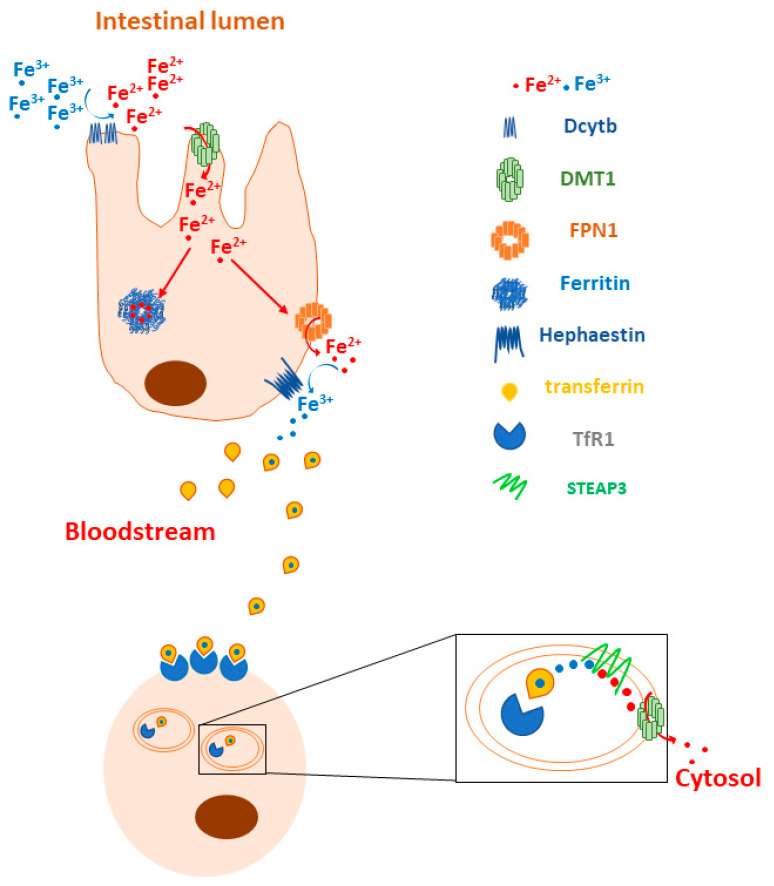
Overview of iron metabolism. Dietary Fe^3+^ is reduced to Fe^2+^ by DCYTB, which is located at the apical membranes of enterocytes facing the intestinal lumen. DMT1 mediates the entry of reduced iron into the cells, where it is stored in ferritin, used in various metabolic reactions or delivered to the basolateral membrane for FPN1-mediated release into the bloodstream. Hp oxidizes Fe^2+^ to Fe^3+^, which can be bound by the iron carrier Tf. Cells bind holo-transferrin through TfR1 on their cell surface and internalize this complex via clathrin-dependent endocytosis. Finally, acidic dissociation of Fe^3+^ occurs into endosomes, followed by a STEAP3-mediated reduction to Fe^2+^, which is released into the cytosol via DMT1.

Iron metabolism is regulated at different levels in human cells. A crucial step is the reduction of dietary iron Fe^3+^ to Fe^2+^ before its uptake into enterocytes, and this process is mediated by the iron-reducing ferric reductase duodenal cytochrome B (DCYTB) at the apical membranes of the enterocytes facing the intestinal lumen. The reduced iron then enters the cells across the divalent metal transporter 1 (DMT1), where it is (i) used for specific metabolic reactions, (ii) stored in ferritin or (iii) delivered to the basolateral membrane for ferroportin (FPN1)-mediated release into the bloodstream. Then, ferrous iron is oxidized to Fe^3+^ by hephaestin (Hp) and bound to transferrin (Tf), the main natural iron carrier in plasma. Under normal physiological conditions, erythroblasts, hepatocytes and macrophages bind the transferrin–iron complex on their cell surfaces via transferrin receptors 1 and 2 (TfR1 and TfR2) and internalize it via clathrin-dependent endocytosis. Then, the acidic dissociation of iron Fe^3+^ occurs into endosomes, followed by a ferrireductase six-transmembrane epithelial antigen of prostate (STEAP3)-mediated reduction into Fe^2+^_,_ which is released to the cytosol via DMT1. Under physiological conditions, it will constitute the “labile iron pool (LIP)” and will be either incorporated into heme (mainly erythroblasts) or stored in ferritin (erythroblasts, hepatocytes and macrophages) (Figure 1) [5].

**Figure 2:**
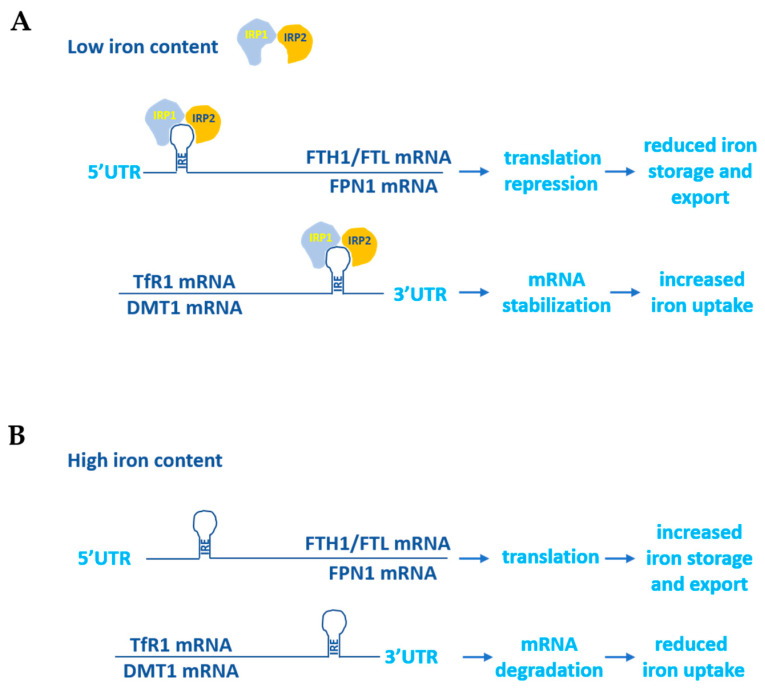
IRP1- and IRP2-dependent control of iron metabolism genes’ mRNAs. (**A**) Under iron-depleted conditions, IRP1 and IRP2 proteins bind to IREs located at the 5′ and 3′UTRs of target genes. The binding of IRPs to the 5′UTR IREs induces the translational repression of ferritin and FPN1 genes, whereas their binding to the 3′UTR IREs stabilizes the mRNAs of TfR1 and DMT1. These coordinated actions decrease iron storage and enhance iron uptake in the cells. (**B**) Under iron-rich conditions IRP1 and IRP2 do not bind IREs, allowing for ferritin and FPN1 translation and the degradation of TfR1 and DMT1 mRNAs.

Given that the excess and lack of iron are two potentially dangerous conditions for the human body due to the ability of iron to contribute substantially to the regulation of the cellular redox state and proper erythropoiesis, its homeostasis is tightly controlled. To this end two iron-dependent proteins, iron-responsive element binding proteins (IRPs) 1 and 2, bind to the iron-responsive elements (IREs) located at the 5′ and 3′UTRs of the main iron metabolism genes’ mRNAs. The binding of the IRPs to the 5′UTR IREs determines translational repression, while their binding to the 3′UTR IREs stabilizes the mRNAs and increases protein synthesis. For example, when the cellular iron concentration is high, IRPs do not bind to either the 5′UTR IREs of FPN1 and ferritin mRNAs, thus allowing their translation, or to the 3′UTR IREs of DMT1 and TfR1, thus allowing their degradation. On the contrary, in the case of an increased demand for cellular iron uptake, the binding of IRPs to the corresponding IREs located at the 5′ and 3′UTRs of the same mRNAs leads to the inhibition of FPN1 and ferritin synthesis and the stabilization of the DMT1 and TfR1 transcripts (Figure 2) [6,7].

**Figure 3:**
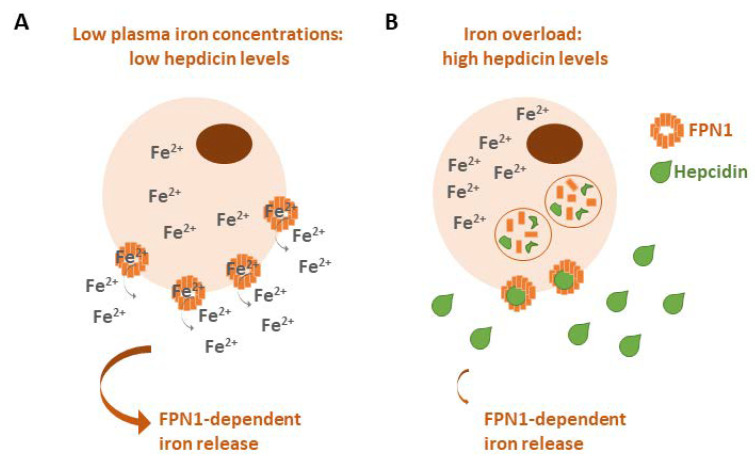
The FPN1/hepcidin axis and systemic plasma iron homeostasis. (**A**) Under iron-depleted conditions hepcidin is downregulated in order to allow for cellular iron release through FPN1. (**B**) Iron overload upregulates the expression of hepcidin, which binds to FPN1 at the cell surface. Hepcidin and FPN1 are internalized and degraded in lysosomes, thus inhibiting iron release.

In addition to these mechanisms, iron homeostasis is balanced by the FPN1/hepcidin axis. Iron overload conditions increase the expression of hepcidin, which binds to FPN1 to promote its internalization and lysosomal degradation to prevent the cellular export of iron, whereas in the case of a lack of iron, hepcidin expression is downregulated to allow for cellular iron release (Figure 3) [8]. The dysregulation of hepcidin could lead to hemochromatosis (iron accumulation) or to anemia (iron deprivation) [9,10].

In addition to the above-described proteins, which are mainly involved in the control of iron metabolism, other regulators have been proposed to play a role in iron homeostasis, such as mitoferrin, or have been ascertained to be pivotal for cellular iron management, such as neutrophil gelatinase-associated lipocalin (NGAL). Mitoferrin, also called SLC25A37, controls the balance of iron between the cytoplasm and mitochondria. Little is known about the regulation of mitoferrin expression: it is still unclear if it is regulated at the transcriptional, translational or post-translational level. Also, how iron influences its activity is still debated. Since mitochondrial dysfunction and disrupted iron homeostasis are associated with a number of pathologic conditions, including atherosclerosis, type 2 diabetes, neurodegenerative diseases and cancer progression, it has been postulated that dysregulated mitoferrin could be found in different diseases. Accordingly, altered expressions of mitoferrin have been reported in in vitro and in vivo models of atherosclerosis, Huntington’s disease, head and neck cancer and hepatocarcinoma [11]. 

**Figure 4:**
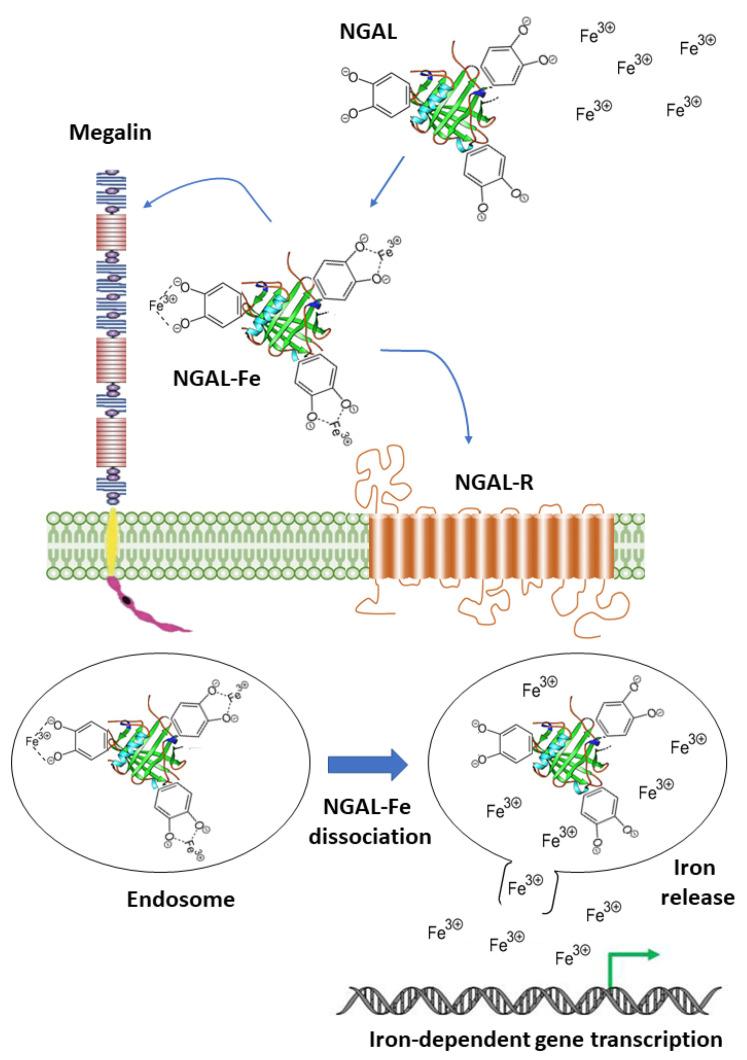
NGAL’s contribution to cellular iron metabolism. The NGAL-siderophores complex (NGAL) binds to extracellular iron (Fe^3+^) (NGAL-Fe) and is delivered inside cells through Megalin or NGAL receptors (NGAL-Rs). Internalized NGAL-Fe undergoes endosome-mediated dissociation to allow for the release of iron into the cytosol, which will be used to activate the transcription of iron-dependent genes.

NGAL (also called Lipocalin-2) is an acute phase protein abundantly secreted by neutrophils during bacterial infections to limit the availability of iron for bacterial growth [12]. It also contributes to the regulation of iron homeostasis in mammalian cells via the SLC22A17- and/or Megalin-mediated uptake of iron-loaded catecholate siderophores to activate the expression of iron-responsive genes like ferritin and transferrin receptor [13,14] (Figure 4). Notably, NGAL can also bind intracellular iron-loaded siderophores and transfer them to the extracellular milieu, thereby decreasing the intracellular iron concentration [15]. In addition to its role as an iron carrier, NGAL binds to matrix metalloproteinase-9 (MMP-9) to protect it from auto-degradation [16].

Although iron plays a beneficial role in many aspects of cellular physiology, it can become toxic in the presence of hydrogen peroxide (H_2_O_2_) by producing a large number of free radicals. The Fenton reaction is a typical example of how iron can play a dangerous role in the cell: when iron Fe^2+^ reacts with H_2_O_2_, it generates the hydroxyl radical (OH·), one of the powerful reactive oxygen species (ROS), and is oxidized to Fe^3+^. The H_2_O_2_ reduces the Fe^3+^ to Fe^2+^, generating the hydroperoxyl radical (OOH·), an additional powerful ROS [17]. These free radicals, if not inhibited by antioxidants, give rise to oxidative stress and react with proteins, lipids, nucleic acids and carbohydrates, causing cellular damage and tissue injury. One of the most common consequences of this oxidative damage is represented by massive lipid peroxidation that causes dramatic modifications in cellular membrane structures, resulting in an iron-dependent non-apoptotic cell death called ferroptosis [18]. Ferroptosis is triggered by the inability of the antioxidant glutathione (GSH) to either counteract the accumulation of ROS, which is determined by cellular iron excess, or to enhance the activity of phospholipid peroxidase GPX4, which prevents the accumulation of peroxidized lipids. GSH failure mainly occurs as a consequence of missed cysteine uptake in its oxidized form (cystine) via the Xc– cystine/glutamate antiporter [19]. Ferroptosis has been found to be associated with different human disorders such as cardiovascular diseases [20], neurodegeneration [21] and cancer [22]. 

### 1.2. Iron in Cancer and Senescence 

In recent years, a substantial body of literature has highlighted the pivotal role of iron metabolism in cancer. It has been largely demonstrated that neoplastic cells need iron to accelerate their proliferative rate, circumvent pro-apoptotic signals, enhance the metastatic spread, shape a tumor-desirable microenvironment and sustain the activity of cancer stem cells [23,24,25,26,27]. The intracellular accumulation of iron is also a distinctive alteration in cellular senescence. 

Cellular senescence is a state of irreversible cell cycle arrest that ensues in cells in response to different types of stress or arises as a conserved developmental program during mammalian embryogenesis [28]. Senescent cells are characterized by distinctive phenotypic alterations, such as a flat and enlarged morphology, the increased expression and activity of lysosomal beta-galactosidase (referred to as senescence-associated beta-gal or SA-β-gal) [29] and the SASP (senescence-associated secretory phenotype), a temporally regulated secretion of cytokines, growth factors and extracellular remodeling enzymes which mediate both the physiological and pathological effects of senescent cells [30,31]. Senescent cells and their secretome contribute critically to organismal aging. Senescent cells have been shown to accumulate in aged tissue and in age-related diseases, where they impair tissue repair and promote tissue dysfunction [32]. Pre-malignant and malignant cancer cells can also undergo senescence and accumulate in pre-neoplastic lesions and in neoplastic tissue from patients with cancer [33]. In preneoplastic lesions, the improper activation of oncogenes induces oncogene-induced senescence (OIS), which acts as a barrier against transformation [33]. Notably, the SASP factors expressed during OIS promote paracrine senescence in adjacent cells, also limiting tumorigenesis [34]. Finally, a senescent-like phenotype indicated as therapy-induced senescence (TIS) can be induced in neoplastic cells via treatment with conventional and targeted anticancer drugs [35,36,37]. Although both short-term OIS and TIS are beneficial in that they limit the proliferation of pre-neoplastic and neoplastic cells, respectively, the detrimental effects of senescent cells mediated by the SASP prevail in the long term [38]. 

The development of a senescent phenotype is associated with specific metabolic changes, as recently reviewed in [39,40]. In particular, alterations in lipid metabolism have been described. Some alterations, such as the upregulation of the β-oxidation of fatty acids, are necessary for the secretion of SASP factors, whereas others, such as the synthesis of prostaglandin, have been functionally linked to the reinforcement of the senescent cell cycle arrest [39]. Alterations in autophagy have also been observed, with both reduced macroautophagy and selective protein degradation via microautophagy. an increase in lysosomal mass is responsible for increased SA-β-gal, whereas increased lysosomal permeability determines the upregulation of glutaminolysis [39]. In addition, mitochondrial alterations, such as an increase in mitochondrial mass, reduced oxidative phosphorylation and elevated ROS production, have been involved in cellular senescence [40]. Interestingly, senescent cells accumulate transition metals, including manganese, zinc and iron [39]. 

A critical aspect of cellular senescence is the significant heterogeneity of senescent cells. An analysis of senescence-specific gene expression showed dramatically different profiles between different cell types, such as fibroblasts and epithelial cells [41], and different senescence inducers [42]; even within a homogeneous cell population, senescent cells display more variability in mRNA levels than quiescent cells [43]. 

Experimental data have demonstrated that intracellular iron content increases during replicative and stress-induced senescence in normal cells [44,45]. Accordingly, iron accumulation in tissue has been associated with aging and with the development of age-related diseases [46,47]. Although alterations in proteins involved in iron metabolism have been reported in senescent cells, the role of iron in senescence has not been clarified. 

This review presents a protein-by-protein description of iron metabolism alterations in cancer and senescence from a cellular perspective. To this end, we focus on a subset of proteins involved in iron homeostasis whose roles in cancer and senescence have been investigated in a cellular context.

## 2. Key Iron Regulatory Proteins in Cancer and Senescence

### 2.1. Transferrin Receptor 1 and 2 (TfR1, TfR2)

Tf binds circulating iron from plasma in vivo or from milieu in vitro and delivers it to cells via TfR1/TfR2-mediated uptake, followed by clathrin-mediated endocytosis. Hence, TfR1 and TfR2 are key regulators of cellular iron import and iron content [48].

TfR1 is overexpressed on the surfaces of many different types of cancer cells [49], to the degree that it has been identified as a universal cancer marker [50]. Several studies have shown that tumor cells express higher levels of TfR1 than normal cells to increase the intracellular iron uptake needed to trigger the different metabolic pathways involved in different aspects of cancer development. Interfering with TfR1 expression or activity suppresses the proliferation, survival, migration and invasion of cancer cells from different human tumors [51]. Generally, the increased expression of TfR1 in malignant cells correlates with an aggressive tumor phenotype, and it is predictive of a poor prognosis in a number of malignancies of both solid and hematopoietic origins [48]. Evidently, cancer cells have every interest to induce unregulated TfR1 expression which, on the contrary, is tightly controlled in normal cells, as described above. To this end, aside from the well-known genes involved in the regulation of TfR1 expression, such as IRP1 and IRP2, different pro-tumorigenic proteins positively influence TfR1 expression in cancer cells, including the proto-oncogene c-MYC, which binds to a conserved E-box binding site of the TfR1-encoding gene promoter [52], hypoxia-inducible factor 1 (HIF-1), which activates the expression of TfR1 under iron-deficient conditions by binding to an upstream hypoxia response element [53], and 17β-estradiol, which induces the expression of TfR1 in estrogen receptor-positive breast cancer [54]. Moreover, it has been shown that TfR1 overexpression warrants robust iron uptake in breast carcinoma cells to protect neoplastic cells from the cytolytic activities of natural killer (NK) cells [55] and that TfR1 is able to mediate NF-κB activation in tumor cells by interacting with the inhibitor of the NF-κB kinase (IKK) complex, leading to an increased survival of malignant cells [56]. Interestingly, an elevated expression of TfR1 in breast cancers and gliomas correlates with an adverse tumor microenvironment that mainly constitutes infiltrating pro-tumorigenic immune cells [57,58]. Finally, TfR1 mediates iron accumulation in hepatocarcinoma-associated cancer stem cells (CSC) to contribute to the retention of their stemness, thus promoting cancer progression [59]. The elevated expression, surface localization and pro-tumorigenic activity of TfR1 in neoplastic cells have made it a promising target for anti-cancer therapy. A common strategy is represented by the use of either Tf or specific anti-TfR1 antibodies to promote the delivery of anti-cancer drugs such as chemotherapeutics, toxins and oligonucleotides [60]. Alternatively, one could take advantage of the ability of anti-TfR1 antibodies to directly inhibit TfR1 activity or induce antibody-mediated effector functions such as antibody-dependent cell-mediated cytotoxicity (ADCC), antibody-dependent cell-mediated phagocytosis (ADCP) and complement-dependent cytotoxicity (CDC) [61].

TfR2 is highly expressed in the liver, and its expression is not regulated by intracellular iron levels but seems to be influenced by the cell cycle [62]. TfR2 is upregulated in tumor cell lines from ovarian cancer, colon cancer and glioblastoma, and to a lesser extent in tumor cell lines from leukemia and melanoma [63]. Interestingly, in these cells, TfR2 expression is inversely correlated to the expression levels of TfR1 and c-myc, and it is lowered by c-myc upregulating agents such as iron, while it is enhanced by c-myc downregulating molecules such as iron chelators [64]. Colon carcinomas and glioblastomas frequently express Tfr2: it has been reported that TfR2 is detectable in about 26% of cases of colon cancer examined; it is not related to histological grade, and it is preferentially expressed during the S-M phases of the cell cycle. Similarly, a strong degree of TfR2 upregulation has been found in glioblastomas, where TfR2 levels correlate with an overall longer survival for patients previously treated with radiotherapy and temolozomide [65].

Senescence-associated alterations in TfR1 have been described in human diploid fibroblasts (HDF) exposed to sub-lethal concentrations of tert-butyl-hydroperoxide or ethanol which display features of premature senescence. In these stress-induced senescent fibroblasts, an upregulation of transferrin receptor has been detected via differential display RT-PCR. The upregulation of TfR1 has been confirmed in replicative senescent fibroblasts compared to young, proliferating cells [66]. Elevated levels of TfR1, associated with an increase in intracellular iron content, have been also detected in mouse embryonic fibroblasts (MEFs) induced to undergo senescence via sublethal gamma irradiation when compared to primary non-irradiated MEFs [45]. The acquisition of a senescent phenotype is a gradual process. Interestingly, through analyzing the temporal development of senescence in irradiated MEFs, these authors show that intracellular iron content gradually increases, reaching a plateau in fully senescent MEFs. TfR1 protein levels also gradually increase, paralleling the amount of intracellular iron. Hence, in these cells, the progressive accumulation of intracellular iron content during senescence correlates with a progressive upregulation of TfR1, suggesting a gradual increase in iron uptake from the extracellular milieu.

A similar link between cellular senescence, iron uptake and TfR1 expression has been suggested in chronic obstructive pulmonary disease (COPD) [67]. COPD is a condition of accelerated lung aging that is characterized by chronic inflammation and the accumulation of senescent cells in the lung. Increased levels of TfR1 have been detected in small airway epithelial cells from COPD patients. The uptake of iron is also increased in COPD small airway epithelial cells when compared to small airway epithelial cells from non-smokers, and the intracellular iron content is also increased. In line with an increased iron uptake, lung homogenates from COPD patients demonstrate increased protein levels of TfR1, which correlates with the expression of senescence markers. Finally, the in vitro induction of therapy-induced senescence (TIS) in small airway epithelial cells via treatment with doxorubicin confirms an enhanced expression of both senescence markers p21Cip1 and TfR1, further linking senescence and elevated iron uptake via TfR1 [67].

Senescent cells can spread the senescent phenotype to neighboring proliferating cells via secreted SASP (senescence-associated secretory phenotype) factors, a process referred to as paracrine senescence [68]. Admasu and colleagues have analyzed both primary endothelial cells induced to undergo TIS with doxorubicin and DPP4-positive paracrine senescent endothelial cells. Both TIS and paracrine senescent endothelial cells accumulate intracellular ferrous iron Fe^2+^, but the iron importer TfR1 is found to be downregulated, suggesting that in this cell system, increased iron uptake is not responsible for the elevated intracellular iron levels [69]. The downregulation of TfR1 has also been described in senescent cancer cells. Colorectal cancer cells (CRC) HCT116, SW620 and SW480 which have been induced to undergo senescence via treatment with either doxorubicin or H_2_O_2_ demonstrate reduced TfR1 levels and elevated levels of labile ferrous iron [70].

Overall, these data indicate that TfR1 is able to meet the metabolic needs of cancer cells by acting as an essential regulator of iron uptake, representing a crucial molecule for cancer development and progression. Its expression is also modulated in different types of senescence, but senescence-induced alterations in TfR1 levels do not necessarily reflect the intracellular iron content. 

### 2.2. Ferritin

Ferritin is a protein complex composed of two subunit types, ferritin light chain (FTL) and ferritin heavy chain (FTH1), and represents the major iron storage protein in mammals. Ferritin plays a key protective role in cells by storing excess iron in a redox inactive form (Fe^3+^), thereby preventing iron- and ROS-dependent damage to cellular components and ferroptosis. The extraction and utilization of ferritin-bound iron under iron-depleted conditions mostly depends on the lysosomal degradation of ferritin, a process known as ferritinophagy [71]. 

Ferritin modulates the proliferation, apoptosis and invasion of cancer cells [72]. Therefore, its levels are dysregulated in many human tumors, such as breast cancer [73], prostate cancer [74] and glioblastoma [75], and are associated with poor survival rates in multiple cancers [72]. The enhanced expression of FTH1 in HCCLM3 and MHCC97H hepatocarcinoma cells leads to increased cell proliferation via the downregulation of H_2_O_2_ and ROS levels [76]. Elevated FTL contributes to glioblastoma cells’ growth via the inhibition of the GADD45a/JNK pathway and the upregulation of c-MYC and cyclin D1 [77], while FTL downregulation decreases the proliferation of ACC-MESO-1 and CRL-5915 mesothelioma cells [78]. In addition, tumor-associated macrophages (TAMs) stimulate the proliferation of cancer cells through ferritin secretion in the tumor microenvironment, highlighting that not only intracellular but also extracellular ferritin could contribute to neoplastic growth [79,80]. Interestingly, the ability of ferritin to sustain the growth of neoplastic cells is independent of its iron content: for instance, in MCF7 and T47D breast cancer cells, ferritin drives proliferative activity in an iron-independent fashion [73]. While the role of ferritin in the positive regulation of cell proliferation in tumors is robustly established, its ability to protect cancer cells from apoptosis seems to be context-dependent, even if in the majority of cases, it acts to promote the survival of neoplastic cells. For instance, in H460 and A549 non-small cell lung cancer (NSCLC) cells, FTH1 activates the apoptotic pathway via the upregulation of p53 [81], and in erythroleukemic K562 cells, mitochondrial ferritin promotes cell death via iron sequestration and JAK/STAT5 activation [82]. On the other hand, blocking FTH1 in MCF7 breast cancer cells increases their susceptibility to apoptosis [83], whereas its overexpression rescue HeLa cells from TNFα-induced cell death [84]. Similarly, FTL inhibition in melanoma cells determines increased sensitivity to apoptosis and reduced growth rate [85]. As in the case of its pro-proliferative activity, the pro-survival activity of ferritin is also independent of cytosolic iron content but could be exerted via the direct inhibition of pro-apoptotic proteins: FTH1’s association with the pro-apoptotic BAX protein protects 293T cells from BAX-mediated cell death [86]. A role of ferritin has also been demonstrated in cancer progression, particularly for its ability to interfere with two critical prerequisites for the invasion and spread of cancer cells: the epithelial mesenchymal transition (EMT) and angiogenesis. Ferritinophagy in A549 cells enhances a TGFβ-induced EMT [87], and FTH1 silencing in MCF7 and H460 cells activates either CXCR4/CXCL12 axis or iron-dependent oxidative stress to foster the EMT [88]. Accordingly, a decrease in ferritin in TfR1-silenced breast cancer cells is accompanied by an increase in vascular endothelial growth factor (VEGF), a well-known pro-angiogenic factor, through the stabilization of the hypoxia-induced factor (HIF)-1α transcription factor, whose expression is enhanced via oxygen starvation (hypoxia) to promote angiogenesis [89]. On the contrary, in gliomas, angiogenesis is induced by mitochondrial ferritin overexpression, which activates the small nucleolar RNA host gene 1 (SNHG1) to stimulate endothelial cells [90]. 

High ferritin levels have been found in CSCs associated with cholangiocarcinoma [91], glioblastoma [75], lung cancer [92], ovarian cancer [93] and breast and prostate cancers [94]. This overexpression correlates with the ability of CSCs to form tumor spheres in vitro and in vivo and is linked to a poor prognosis given that the activity of CSCs in the tumoral mass, which is well established, is responsible for relapse and progression. 

Finally, ferritin has been shown to be involved in the resistance of neoplastic cells to anti-cancer treatments (chemotherapy, radiotherapy, immunotherapy and targeted therapy) due to its antioxidant features, which allow tumor cells to survive the stresses exerted by therapies. The elevated expression of both FTH1 and FTL has been reported to correlate with chemo- and radio-resistance in cancer cell lines derived from different types of human tumors, including leukemia [95,96], melanoma [85], glioma [97,98], breast cancer, bladder cancer, lung cancer and prostate cancer [99,100].

Elevated levels of ferritin have been detected in various models of cellular senescence. For instance, replicative and stress-induced WI-38 fetal lung HDFs show increases in steady-state levels of ferritin light chain (FTL) transcript when compared to proliferating cells [66]. Masaldan describes an up to 10-fold increase in the ferritin protein amount in replicative and irradiation-induced senescent MEFs. Similar ferritin elevations have also been detected in primary HDFs as well as prostate epithelial cells undergoing replicative or IR-induced senescence. In line with the upregulation of this iron storage protein, all the senescent cells analyzed, irrespective of the senescence-inducing stimulus, demonstrate significant intracellular iron accumulation. Importantly, increased ferritin levels in senescent cells have been shown to be related to an impaired ferritinophagic flux due to a senescence-dependent autophagic/lysosomal dysfunction [45]. 

A crucial role of the autophagy–lysosome pathway in regulating ferritin turnover in senescence has been also demonstrated in replicative senescent human dermal fibroblasts, in which the relative contributions of the two major proteolytic systems, i.e., the ubiquitin–proteasome system and autophagy–lysosome system, to ferritin degradation have been investigated. Ferritin heavy chain (FTH1) displays a higher steady-state level and a reduced protein turnover in the senescent cells, and impaired autophagy is responsible for reduced FTH1 degradation in senescent cells [101]. A senescence-specific reconfiguration of the lysosomal compartment has been recently described in SK-MEL-103 melanoma cells induced to undergo TIS by treatment by the CDK4/6 inhibitor palbociclib [102]. SK-MEL-103 senescent cells display not only a well-described increase in lysosomal mass [103], but also distinctive alterations in the compositions and amounts of both resident lysosomal proteins and lysosomal substrate proteins. In particular, proteomic analyses of lysosomes isolated from SK-MEL-103 TIS cells have allowed for the identification of proteins whose degradation rates are specifically decreased in senescence, including FTH1 and FTL. Furthermore, FTH1 protein levels are specifically increased in both total cell lysates and in the conditioned media from TIS cells when compared to proliferating SK-MEL-103 [102]. Hence, FTH1 accumulates in these senescent cancer cells and appears to be subjected to lysosomal secretion upon the fusion of lysosomes with the plasma membrane. Interestingly, cellular senescence induced via various stimuli in normal or neoplastic cells has been associated with increased biogenesis and an increase in the release of extracellular vesicles [104], and iron-loaded ferritin is known to be secreted via exosomes [105]. It is plausible to hypothesize that lysosomal secretion and the exososomal release of ferritin might both represent means of excreting excess iron from senescent cells, but a role for the elimination of ferritin in senescent cells, as well as its potential effects on the microenvironment, has not been clarified.

Interestingly, Masaldan and colleagues demonstrated that the co-treatment of primary MEF with the iron chelator deferiprone and irradiation inhibits both iron and ferritin accumulation but does not affect senescence development. These results suggest that the acquisition of a senescent phenotype precedes intracellular iron accumulation and that iron accumulation is not required for cellular senescence [45]. Additional evidence that iron accumulation ensues after senescence and is not an upstream contributor event has been provided in H_2_O_2_-induced senescent CRCs [70]. In these cells, pretreatment with iron chelator desferoxamine (DFO) followed by hydrogen peroxide exposure does not prevent the acquisition of a senescent-like phenotype. In this study, however, senescent cancer cells displayed reduced amounts of FTH1 and, accordingly, accumulated intracellular iron in the form of ferrous iron. In line with these observations, the overexpression of FTH1 attenuates Fe^2+^ accumulation. These data suggest that FTH1 downregulation can contribute to the accumulation of redox-active ferrous iron in senescent CRC cells, likely promoting ferroptosis. These authors also uncovered a novel role of ribosomal L1 domain-containing 1 (RSL1D1) RNA-binding protein as a direct regulator of FTH1. In particular, RSL1D1 binds to the 3′-untranslated region of FTH1 and promotes FTH1 mRNA stability. Accordingly, RSL1D1 knockdown in senescent CRC cells decreases FTH1 levels and induces the intracellular accumulation of ferrous iron, thereby sensitizing cells to ferroptosis. Hence, ferritin is implicated in modulating the cellular labile iron pool and the susceptibility to ferroptosis in senescent cancer cells. 

A protective role of ferritin in limiting labile ferrous iron-dependent ferroptosis has also been demonstrated in a model of premature senescence induced via bleomycin in HDFs [106]. These senescent cells showed elevated levels of ferritin heavy and light chain. Interestingly, treatment with the BET family inhibitor JQ1 results in decreased levels of mRNA expression of both FTH1 and FTL and activates ferroptosis selectively in senescent HDFs, not affecting proliferating control cells. The ferroptosis resistance genes BRD4, GPX4, SLC7A11 and Nrf2 are also selectively downregulated by JQ1 in senescent cells. 

Non-ferritin-bound iron can induce not only ferroptosis but also premature senescence, as demonstrated in a cell model of neuroferritinopathy (NF), a rare autosomal dominant disease caused by mutations in the FTL gene. Patient-derived fibroblasts, neural progenitors and neurons exposed to Fe-ammonium citrate to generate iron overload develop a senescent-like phenotype, while control cells expressing FTL are unaffected [107]. These data further support the protective role of ferritin and suggest the direct involvement of ferrous iron in the aging process. 

Overall, these studies indicate that ferritin, given its multifaceted roles in iron-related tumor biology, strongly supports the proliferation, survival, invasion, therapy resistance and relapse of cancer cells. Senescent cells can accumulate iron as either harmful labile ferrous iron [67,70] or as redox-inactive ferritin-bound Fe^3+^ [45,102], but the mechanisms determining such differences are still unclear.

### 2.3. Ferroportin (FPN1)

FPN1 is a transmembrane exporter essential for the release of ferrous ion (Fe^2+^) from cells. Its expression is strongly downregulated in human tumors such as those found in ovarian cancer [93], prostate cancer [108], breast cancer [109] and lung cancer [110], very likely because cancer cells have an interest in preserving intracellular iron content to achieve all the pro-tumoral activities in which iron metabolism is involved. Conversely, hepcidin expression is increased in neoplastic cells to ensure a high rate of FPN1 degradation [108,111,112]. The role of FPN1 in cancer has been studied in cancer cell lines from different malignancies: decreased FPN1 levels following the upregulation of ferritin lead to the enhanced proliferation, survival and migration of prostate cancer cells, whereas the overexpression of FPN1 stimulates autophagy and reduces cancer cells’ oncogenic potential [113]. In breast cancer cells, FPN1 interferes with tumor growth and progression [114,115], and in thyroid cancer cells, its degradation, which is mediated by secreted hepcidin, promotes cell proliferation [116]. Interestingly, FPN1 inhibition makes neuroblastoma and lung cancer cells more sensitive to erastin-induced ferroptosis [117,118].

Both deregulated expression and altered subcellular localization have been described for FPN1 in senescent cells. For instance, in irradiation-induced senescent MEFs, increased FPN1 protein levels were detected, not at the plasma membrane but mislocalized intracellularly, thereby suggesting the accumulation of an inactive transporter. Since FPN1 is degraded through the autophagic pathway, the observed intracellular accumulation has been linked to the previously described senescence-dependent dysfunction of the autophagic/lysosomal pathway [45]. In contrast, in primary endothelial cells induced to undergo TIS and in paracrine senescent endothelial cells, FPN1 protein levels are reduced when compared to non-senescent cells. The role of FPN1 in this cell system has not been fully analyzed, but both primary and paracrine senescent endothelial cells accumulate high levels of ferrous iron; therefore, a reduction in iron efflux via the downregulation of FPN1 might represent a plausible mechanism for this accumulation [69]. As previously described, FPN1 downregulation has been observed in multiple cancer types in which it provides a mechanism for the retention of intracellular labile iron. In cancer cells, an elevated Fe^2+^ content is required to sustain enhanced proliferation. Interestingly, in a panel of metastatic head and neck squamous carcinoma cell lines (HNSCCs), conditional FPN1 overexpression not only inhibits proliferation but even induces premature senescence via the loss of labile iron [119]. FPN1 overexpression also induces cell cycle arrest in prostate cancer cells in vitro and in vivo in mouse xenografts. Interestingly, although these authors did not investigate a potential induction of premature senescence in these prostate cancer cell lines, microarray analyses of cells overexpressing FPN1 highlight alterations in SASP genes [113]. 

These data indicate that reducing FPN1 expression in cancers via increasing intracellular ferrous iron may contribute to a highly proliferative and aggressive phenotype, whereas the effects of elevated ferrous iron in senescent cells are less clear. However, the downregulation of FPN1 in cell cycle-arrested senescent cells clearly uncouple Fe^2+^ accumulation from cellular proliferation. This observation is supported by recent data obtained in KRAS-driven tumors. In this study, the acute induction of oncogenic KRASG12D signaling in MEFs and primary dermal fibroblasts induced FPN1 downregulation, labile iron accumulation and the features of oncogene-induced senescence (OIS). Increased intracellular ferrous iron is also observed in immortalized bronchial and pancreatic human epithelial cells after the expression of KRASG12D [120]. Hence, this study demonstrates that dysregulated iron export and sustained intracellular Fe^2+^ accumulation both represent consistent features of KRAS-induced senescence in primary cells. Importantly, the authors also demonstrate that this altered iron metabolic state can be therapeutically exploited. 

Overall, these studies indicate that the functional inactivation of FPN1, via protein downregulation or intracellular delocalization, and the consequent disruption of iron homeostasis characterize various types of cellular senescence. The role of FPN1, if any, in the acquisition and/or the maintenance of a senescent phenotype deserves further investigation.

### 2.4. Neutrophil Gelatinase-Associated Lipocalin (NGAL)

In addition to transferrin, NGAL delivers iron to mammalian cells, thereby contributing to cellular iron homeostasis. NGAL has been found to be overexpressed in a high number of human tumors given its ability to promote cancer development and progression. For this reason, NGAL levels correlate with the aggressive behavior of cancer cells and are associated with poor outcomes of malignancies of either solid or hematopoietic origin. Elevated NGAL expression has been found in breast and colon cancer patients with adverse prognosis [121,122] and in patients with chronic myeloid leukemia (CML), as well chronic lymphocytic leukemia (CLL) [123,124]. NGAL levels are also elevated in early lesions of pancreatic tumors, where they further increase with disease progression [125], and in thyroid carcinomas, especially of the undifferentiated type [126]. In addition, cell lines from endometrial, lung and oral cancers expressing high levels of NGAL show strong resistance to radio- and chemo-therapy [127,128], while in prostate cancer cell lines, the elevated expression of NGAL increases their proliferation and oncogenic potential [129]. NGAL also facilitates the metastatic spread of neoplastic cells due to its ability to preserve MMP-9 enzymatic activity: an increase in NGAL/MMP-9 complexes has been found associated with a higher metastatic risk in patients with breast [130], gastric [131] and endometrial cancer [132], while NGAL knockdown in prostate cancer-derived cell lines [133] or the impairment of NGAL/MMP-9 complex formation in anaplastic thyroid carcinoma-derived cell lines [134] led to a marked decrease in their invasive potential. Finally, since NGAL is also secreted by stromal, immune and inflammatory cells infiltrating tumor masses, it actively participates in the shaping of the tumor microenvironment. The NGAL secreted by N2-type neutrophils and by tumor associated macrophages (TAMs) strongly supports breast cancer cell colonization in the lung [135,136], whereas the inhibition of NGAL in CT26 colon carcinoma cells drastically reduces the number of infiltrating macrophages and lymphocytes in the tumor microenvironment of allografts generated in syngeneic mice [137]. It is important to note that the pro-tumorigenic role of NGAL mainly relies on its iron carrier property and in part, on its ability to enhance MMP-9 activity, since the disruption of siderophores and the binding of MMP-9 to NGAL impair its role in cancer [13]. Interestingly, it has also been shown that NGAL inhibits ferroptosis in CRCs via decreasing intracellular iron levels and stimulating the expression of glutathione peroxidase 4 (GPX4) to prevent membrane lipid peroxidation [138]. Moreover, the nuclear protein 1 (NUPR1)-dependent upregulation of NGAL expression inhibits ferroptosis in pancreatic cancer [139]. Similarly, NGAL-stimulated renal cell carcinoma cells are protected from erastin-induced ferroptosis [140]. Overall, these studies indicate that NGAL plays a pivotal role in cancer, contributing, with other iron-related proteins, to the development of malignant cells’ iron-dependent, pro-tumorigenic functions.

A potential role of NGAL in aging and senescence has been investigated by Bahmani and colleagues, who reported a positive correlation between plasma levels of NGAL and age in a cohort of healthy volunteers classified into three groups of age. Furthermore, these authors found that the expression of NGAL increases in human bone marrow-derived mesenchymal stem cells (MSCs) induced to undergo premature senescence via exposure to oxidative stress, and in this experimental setting, NGAL overexpression counteracts senescence induction. In contrast, no alterations in NGAL expression are observed during replicative senescence, suggesting that NGAL does not play any role in the natural aging process of MSCs [141]. Hence, this study suggests a specific role for NGAL in stress-induced senescence. 

A distinctive trait of senescent cells is the SASP, a temporally regulated secretion of cytokines, chemokines, growth factors and extracellular remodeling enzymes which mediate both the physiological and pathological effects of senescent cells [30,31]. Notably, NGAL has been identified as SASP factor in various models of stress-induced senescence in both normal and neoplastic cells. For instance, the induction of NGAL has been described in CRC cells: HCT116 cells induced to undergo TIS via exposure to either 5-fluorouracil or doxorubicin develop a SASP and secrete NGAL into the medium. Notably, this senescent secretome elicits an epithelial-to-mesenchymal transition and invasiveness in proliferating colon and rectal cancer cell lines [142]. NGAL is also expressed in the SASP of etoposide-treated breast and lung cancer cells [143]. Furthermore, analyses of biopsy samples from breast cancer patients collected prior to or following neoadjuvant chemotherapy treatments demonstrated a significant upregulation of SASP markers, such as IL-6, and NGAL after treatments [144]. Although these authors did not analyze additional markers of premature senescence in biopsy specimens, these data support a TIS-associated increase in NGAL levels in cancer cells. However, in these studies, the specific role(s) and/or effect(s) of NGAL on senescence were not investigated in detail. Interestingly, proliferating breast cancer cells exposed to the secretome from OIS or TIS fibroblasts upregulate NGAL, and NGAL appears to mediate several pro-tumoral effects of the SASP, such as increased proliferation and migration and the epithelial-to-mesenchymal transition [144]. NGAL was also identified as a SASP factor in a mouse model of spinal cord injury. In this latter model, senescent cells developed at the lesion periphery and exerted their pathophysiological effects through the SASP containing NGAL [145]. 

These studies indicate that NGAL is expressed in various SASP programs in both normal and neoplastic cells and particularly in stress-induced senescence, but the specific role of NGAL as part of the SASP and its potential functions in the acquisition and maintenance of a senescent phenotype have not been fully investigated. Notably, NGAL is able to bind intracellular iron-loaded siderophores and mediate their release out of the cells [15]. Therefore, increases in the expression and secretion of NGAL might represent additional means of eliminating excess iron from senescent cells. Finally, it may be worth recalling that NGAL exerts its pleiotropic properties not only through the regulation of iron homeostasis but also via NGAL/MMP-9 complex formation. Hence, whether NGAL functions as iron-binding protein during senescence or if other NGAL functions prevail must be clarified. 

### 2.5. Other Iron Proteins

In addition to those mentioned above, additional iron proteins could play minor roles in cancer, whereas to the best of our knowledge, no data are available for senescence:

- **Duodenal cytochrome B (DCYTB):** a ferrireductase that reduces Fe^3+^ to Fe^2+^ to allow for the uptake of iron by DMT1 on the surfaces of duodenal cells [5]. Lemler and co-workers found that DCYTB expression is a favorable prognostic factor in breast cancer patients because it correlates with a better response to therapy and an increased progression-free survival [146]. Interestingly, they also showed that upregulated DCYTB improves outcomes for breast cancer patients via an iron-unrelated mechanism involving the inhibition of FAK activation and cell adhesion [146].
- **Hepcidin:** a small peptide produced by the liver that is able to induce FPN1 degradation to block iron export from cells [8]. Hepcidin is overexpressed in several human tumors, such as breast, lung and prostate cancers, as well as multiple myeloma, for its property to promote neoplastic growth by increasing iron retention in malignant cells [147]. In a breast tumor microenvironment, cancer-associated fibroblasts stimulated hepcidin expression in breast cancer cells via the production of IL-6 [148], while it has been found that hepcidin expression is associated with immune tumor infiltrates in lung cancer, particularly those constituting B cells, CD4 + T cells, macrophages, neutrophils and dendritic cells [149].
- **Divalent metal transporter 1 (DMT1):** a key protein in the regulation of iron homeostasis for its ability to enable the translocation of Fe^2+^ to the cytosol after iron endocytosis [5]. Blocking DMT1 in colorectal cancer has been shown to suppress cancer progression [26], and using DMT1 inhibitors has been reported to selectively kill iron-addicted cancer stem cells by inducing lysosomal iron overload [150]. Interestingly, DMT1 inhibition promotes ferroptosis in head and neck cancers [151].

## 3. Conclusions

In normal cells, iron homeostasis is finely regulated in order to supply iron for metabolic processes and to avoid iron-dependent ROS accumulation and cellular damage. In this review, we have summarized data regarding iron metabolism-related proteins in cancer cells and senescent cells. These data provide a clear picture of how cancer cells can remodel metabolism in order to increase iron uptake and reduce iron loss. In line, tumor cells express higher levels of TfR1, with several oncogenes directly inducing TfR1 expression. On the contrary, FPN1 expression is strongly downregulated in human tumors. Conclusive evidence points to a pro-tumoral role for ferritin, which sustains the proliferation of cancer cells and protects them from apoptosis. Finally, NGAL is overexpressed and plays multiple roles in cancer cells, as well as in the tumor microenvironment. Hence, the evidence generated in recent years demonstrates that alterations in iron metabolism critically contribute to an aggressive tumor phenotype and a pro-tumoral microenvironment. These observations indicate that iron metabolism and iron metabolism-related proteins represent valuable targets for the development of new cancer therapeutics. Many promising strategies are currently being investigated to interfere with iron homeostasis in cancer cells. One of the most-explored strategies is based on the use of iron chelators such as DFO: these compounds, which are employed to treat disorders of iron overload, are very effective at reducing the intracellular supply of iron; however, they are unfortunately also highly toxic at the doses that are useful for achieving their therapeutic goals in neoplastic cells. In addition, prolonged treatment with iron chelators induces cancer cells to restore iron homeostasis through the upregulation of DMT1 and TfR1, leading to an increase in the intracellular concentration of iron [152]. Targeting TfR1 is another promising strategy under development, as discussed in Section 2.1 of this review, and increasing evidence indicates hepcidin as a target in the metastatic disease state. The use of antibodies that directly or indirectly inactivate hepcidin in cancer cells increased iron serum levels, but their efficacy was time-limited or restricted only to normal cells [153].

This review also suggests a more complex regulation of iron metabolism in senescent cells, either normal or neoplastic. For instance, iron has been shown to accumulate in senescent cells but either as harmful labile ferrous iron or as redox-inactive ferric iron. TfR1 and ferritin levels are clearly modulated during the development of a senescent phenotype, but their expression seems to be context-dependent, increasing in some in vitro models of the cellular senescence and decreasing in other models, and their levels do not necessarily reflect the intracellular iron content. Hence, how these iron-regulatory proteins are regulated in senescent cells and their role(s) are still unclear. In some cases, the processes that govern alterations in iron-regulatory proteins are starting to be understood. For instance, senescence-specific alterations in the autophagy–lysosome pathway, which critically controls protein degradation, likely contribute to the modulation of ferritin in senescence. So, it is tempting to speculate that while iron-related proteins are mainly regulated at the transcriptional level in proliferating cells, they might be regulated via degradation in senescence. In other instances, however, senescent-specific modifications in iron-regulatory proteins still lack an explanation. 

A functional inactivation of FPN1 has also been demonstrated in various models of cellular senescence via protein downregulation or intracellular delocalization. FPN1 inactivation might explain the increases in ferrous iron content observed in some senescent cells. Finally, NGAL has emerged as a critical SASP component, particularly in stress-induced senescence. Interestingly, senescent cells appear to excrete excess iron bound to ferritin via either lysosomal secretion or exosome release. In this context, the secretion of iron-loaded NGAL might represent an additional means of eliminating excess iron.

In conclusion, the data summarized herein highlight important alterations in iron metabolism in cellular senescence which likely reflect the alterations described in organismal aging and in age-related diseases and open therapeutic opportunities for targeting iron metabolism. However, as previously stressed, senescent cells are highly heterogeneous, and the dysregulation of iron metabolism in senescence requires further investigation. A detailed transcriptional and proteomic analysis of iron-related protein expression, as well as a functional characterization of iron metabolism, across different types of senescent cells could be very informative in understanding the role of iron in cellular senescence. Characterizing iron metabolism heterogeneity in senescent cells would also help in the development of new senotherapeutics.

## Data Availability

Not applicable.
